# Specific ablation of mouse *Fam20C* in cells expressing type I collagen leads to skeletal defects and hypophosphatemia

**DOI:** 10.1038/s41598-017-03960-x

**Published:** 2017-06-15

**Authors:** Peihong Liu, Su Ma, Hua Zhang, Chao Liu, Yongbo Lu, Li Chen, Chunlin Qin

**Affiliations:** 1 Department of Periodontics, Harbin Medical University School of Stomatology, Harbin, Heilongjiang 150001 China; 2 Department of Biomedical Sciences, Texas A&M University College of Dentistry, Dallas, Texas 75246 USA; 3 Longjiang Scholar Laboratory, The First Affiliated Hospital of Harbin Medical University, Harbin, Heilongjiang 150001 China

## Abstract

*FAM20C* mutations in humans cause Raine syndrome and our previous studies showed that global inactivation of mouse *Fam20C* led to bone and dental defects. By crossbreeding *2.3* *kb Col 1a1-Cre* mice with *Fam20C* ^*flox/flox*^ mice, we created *2.3* *kb Col 1a1-Cre;Fam20C* ^*foxl/flox*^ (cKO) mice, in which *Fam20C* was inactivated in cells expressing Type I collagen. This study showed that the long bones of cKO mice were shorter and had a lower level of mineralization compared to the normal mice. The collagen fibrils in *Fam20C*-deficient bone were disorganized and thicker while the growth plate cartilage in cKO mice was disorganized and wider compared to the normal mice. The *Fam20C*-deficient bone had a lower level of dentin matrix protein 1, and higher levels of osteopontin and bone sialoprotein than the normal. The blood of cKO mice had an elevated level of fibroblast growth factor 23 and reduced level of phosphorus. These findings indicate that inactivation of *Fam20C* in cells expressing type I collagen led to skeletal defects and hypophosphatemia. The altered levels of dentin matrix protein 1 and osteopontin in *Fam20C*-deficient bone may be significant contributors to the mineralized tissue defects in human patients and animals suffering from the functional loss of FAM20C.

## Introduction

FAM20C is a kinase belonging to the FAM20 family (“family with sequence similarity 20”)^1–4^. *In vitro* studies have shown that FAM20C catalyzes the attachment of phosphates to serine residues in the Ser-X-Glu (S-X-E) motifs of secretory proteins, which include dentin matrix protein 1 (DMP1) and osteopontin (OPN), two members of the “Small-Integrin-Binding LIgand, N-linked Glycoproteins” (SIBLING) family^2–6^. Inactivating mutations in the human *FAM20C* gene cause Raine syndrome, an autosomal recessive disorder that demonstrates a variety of manifestations^7–11^. Some Raine syndrome patients die shortly after birth^7, 12^, while others may live into middle childhood or even middle adulthood^8–10^. The manifestations reported in Raine syndrome patients include generalized osteosclerosis, rickets, osteomalacia, ectopic mineralization of soft tissues, intracranial calcification, microcephaly, cerebellar hypoplasia, pachygyria, exophthalmos, wide fontanelle, midface hypoplasia, cleft palate, low set ears, hypoplastic nose, choanal atresia and dental problems^7–12^.

FAM20C is believed to phosphorylate over a hundred types of secretory proteins^3^. Previously, our group showed that the *Sox2-Cre;Fam20C* ^*flox/flox*^ mice, in which *Fam20C* was inactivated in nearly all the tissues, suffered from hypophosphatemic rickets, along with an elevation of fibroblast growth factor 23 (FGF23) and a reduction of serum phosphorus in the serum^13^. These *Sox2-Cre;Fam20C* ^*flox/flox*^ mice also showed enamel and dentin defects^14^. The phosphorylation levels of non-collagenous proteins including DMP1 and OPN in the bone and dentin of *Sox2-Cre*; *Fam20C* ^*flox/flox*^ mice were lower than in the normal mice^6^.

FAM20C, which is expressed in a variety of tissues, phosphorylates an extremely broad spectrum of secretory proteins^2–6^ and its inactivating mutations in humans cause very heterogeneous manifestations^7–12^. There is a necessity to specifically inactivate this kinase in tissues or organs that express type I collagen and to systematically characterize such tissues or organs. By crossbreeding the *Fam20C* ^*flox*/*flox*^ mice with the transgenic mice expressing Cre-recombinase driven by the *2.3* *kb Col 1a1* promoter, we generated *2.3* *kb Col 1a1-Cre;Fam20C* ^*flox/flox*^ (cKO) mice, in which *Fam20C* was inactivated in the cells expressing Type I collagen. In a previous report, we described the periodontal defects of the cKO mice^15^. In the present study, we analyzed the skeletal tissues in the cKO mice using X-ray radiography, histology, Goldner’s Masson trichrome staining, picro-sirius red staining, immunohistochemistry and Western immunoblotting approaches. We also measured the serum levels of FGF23 and phosphorus in the cKO mice at different ages. We observed that the cKO mice developed skeletal defects and hypophosphatemia, along with altered levels of DMP1, OPN, bone sialoprotein (BSP) and FGF23 in the *Fam20C*-deficient bone.

## Results

### The *2.3* *kb Col 1a1-Cre;Fam20C*^*flox/flox*^ (cKO) mice had growth retardation and skeletal defects

**Figure 1:**
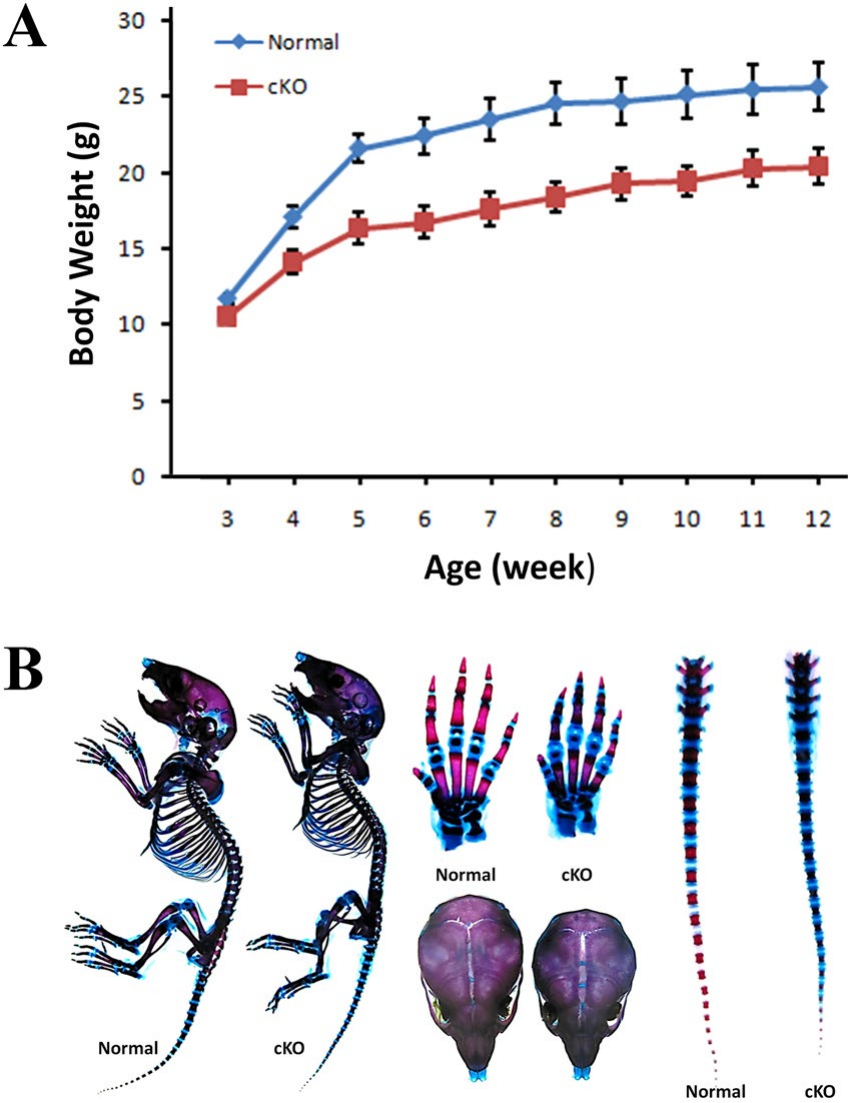
(**A**) Growth curve of cKO mice versus normal mice. At each time point, the body weights were obtained from 10 mice and the average body masses were calculated. At postnatal 3 weeks, the cKO mice were slightly smaller than the normal mice and the differences in body weight between the cKO and normal mice increased with age until postnatal 6 weeks. (**B**) Alizarin red/Alcian blue staining of the skeletons from 1-week-old mice. The skeleton and skull of the cKO mice was smaller. The carpus and tail bones of the cKO mice had more non-mineralized (blue) areas than the normal mice. The fibrous joint (sagittal suture) between two parietal bones was wider in the cKO mice than in the normal mice.

At postnatal 3 weeks, the body weight of the cKO mice was slightly lower than the age-matched normal mice. As the animals aged, the difference in body weight between the cKO and normal mice became more prominent; after postnatal 6 weeks, the difference in body weight between the cKO and normal mice reached a plateau and the similar level of difference remained until postnatal 12 weeks (Fig. 1A). At postnatal 6 weeks, the average body weight of the cKO mice was 16.6 grams while that of the normal mice was 22.3 grams; the body mass of the former was approximately 74.5% of the latter. Overall, the reduction in the body size of the *2.3* *kb Col 1a1-Cre;Fam20C* ^*flox/flox*^ mice was not as remarkable as that of the *Sox-Cre;Fam20C* ^*flox/flox*^ mice in which *Fam20C* was inactivated in nearly all the tissues; the body weight of 6-week-old *Sox-Cre;Fam20C* ^*flox/flox*^ mice was approximately 9 grams, or 40% of the normal mice as previously reported^13^. The Alizarin red/Alcian blue staining of the skeletons from 1-week-old mice revealed that the cKO mice had smaller stature, smaller skulls and delayed closure of cranial sutures, along with delayed ossification in the long bones, carpus and tail bones (Fig. 1B). These observations indicated that inactivation of *Fam20C* in the cells expressing type I collagen resulted in growth retardation and defects in both the axial and appendicular skeletons.

### The long bone of cKO mice had shorter length, lower bone volume and reduced level of mineralization

**Figure 2:**
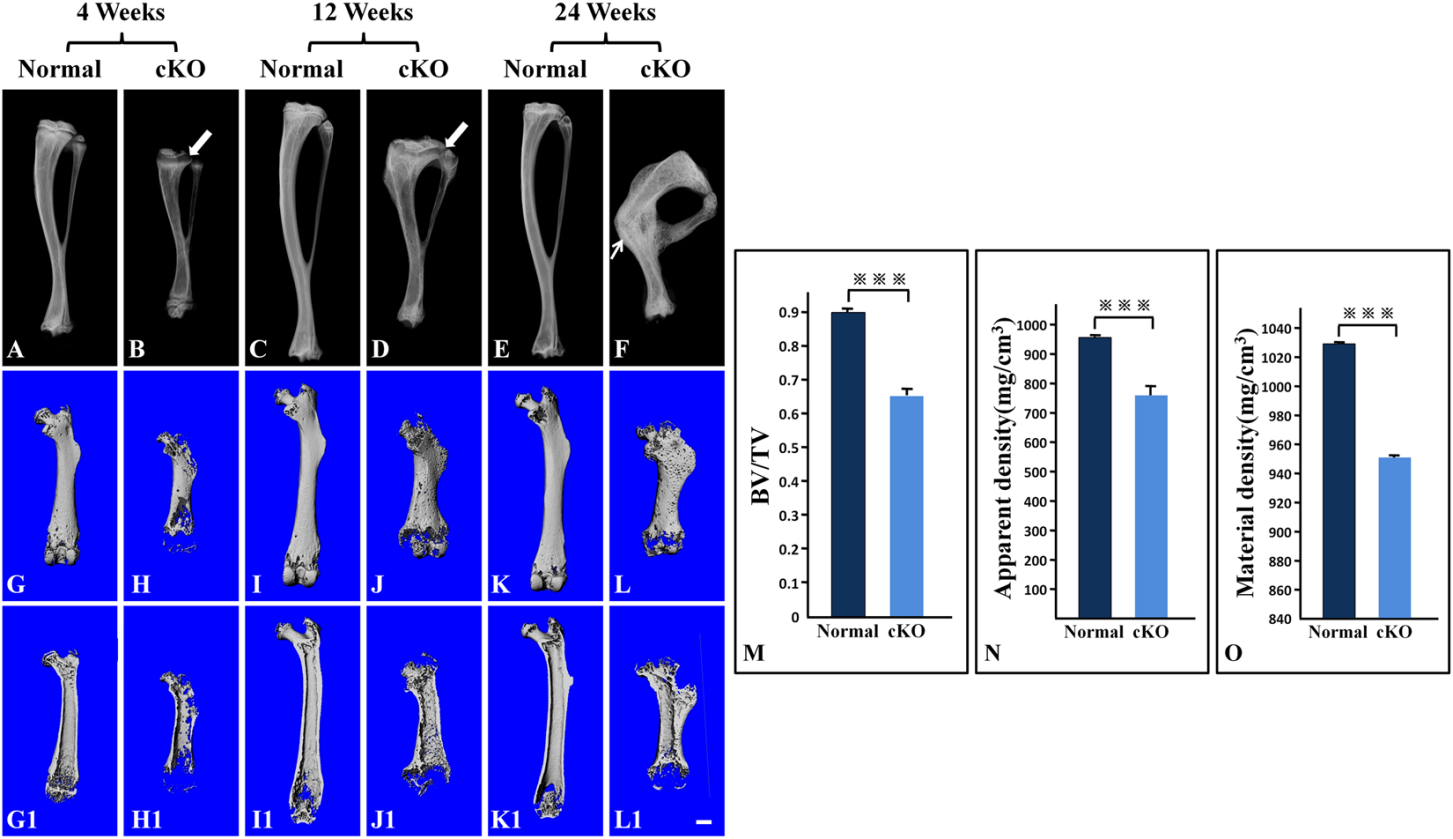
X-ray radiography analyses. (**A**–**F**) Plain X-ray radiographs of mouse tibias; arrows in (**B**) and (**D**) were pointing at the growth plate zones; arrow in (**F**) was pointing at a healed fracture. (**G**–**L**) whole views of µCT scans for mouse femurs; (**G1**–**L1**) longitudinal section views of femurs; (**M**–**O**) quantitative analyses of data acquired from the high-resolution scans of the midshaft regions in the femurs from five mice (n  = 5) at postnatal 12 weeks. Bar = 1.0 mm; ***P < 0.01. The long bones of cKO mice were shorter and had lower radiodensity compared to the age-matched normal mice (**A**–**F**). The growth plate zones (arrows in **B** and **D**) appeared wider in the cKO mice than in the normal mice. The tibia of 24-week-old cKO mice had a fracture (arrow in **F**), which appeared to have healed. The metaphysis regions of the femurs in the cKO mice were enlarged; the outer and inner bone surfaces of the long bones were more porous (**G–L**,**G1–L1**). The cortical bone of cKO mice had significant reductions in BV/TV ratio (**M**), Apparent Density (**N**) and Material Density (**O**).

Plain X-ray examination showed that the long bones of cKO mice were shorter and had a generalized reduction in radiodensity compared to the age-matched normal mice (Fig. 2A,2B,C,D,E,F). The long bones of older cKO mice often had fractures (arrow in Fig. 2F), which further aggravated the deformities of the mouse legs. The μCT analyses demonstrated malformed epiphysis and metaphysis in the long bones of the cKO mice (Fig. 2G,G1,H,H1,I,I1,J,J1,K,K1,L,L1); the outer and inner bone surfaces of *Fam20C-*deficient long bones appeared more porous, which was likely to be a result of increased areas of hypomineralization or the presence of excessive osteoid. The μ-CT quantitative analyses of the midshaft region in 12-week-old mice showed that the cortical bone of cKO mice had significant reductions in the ratio of Bone Volume to Total Volume (BV/TV) (Fig. 2M), Apparent Density (Fig. 2N) and Material Density (Fig. 2O). The BV/TV ratio of cKO mouse bone was 73.8% of the normal mouse bone; the Apparent Density of cKO mice was 80.2% of the normal; the Material Density of cKO mice was 92.7% of the normal.

### Loss of *Fam20C* in type I collagen-expressing cells led to growth plate defects

**Figure 3:**
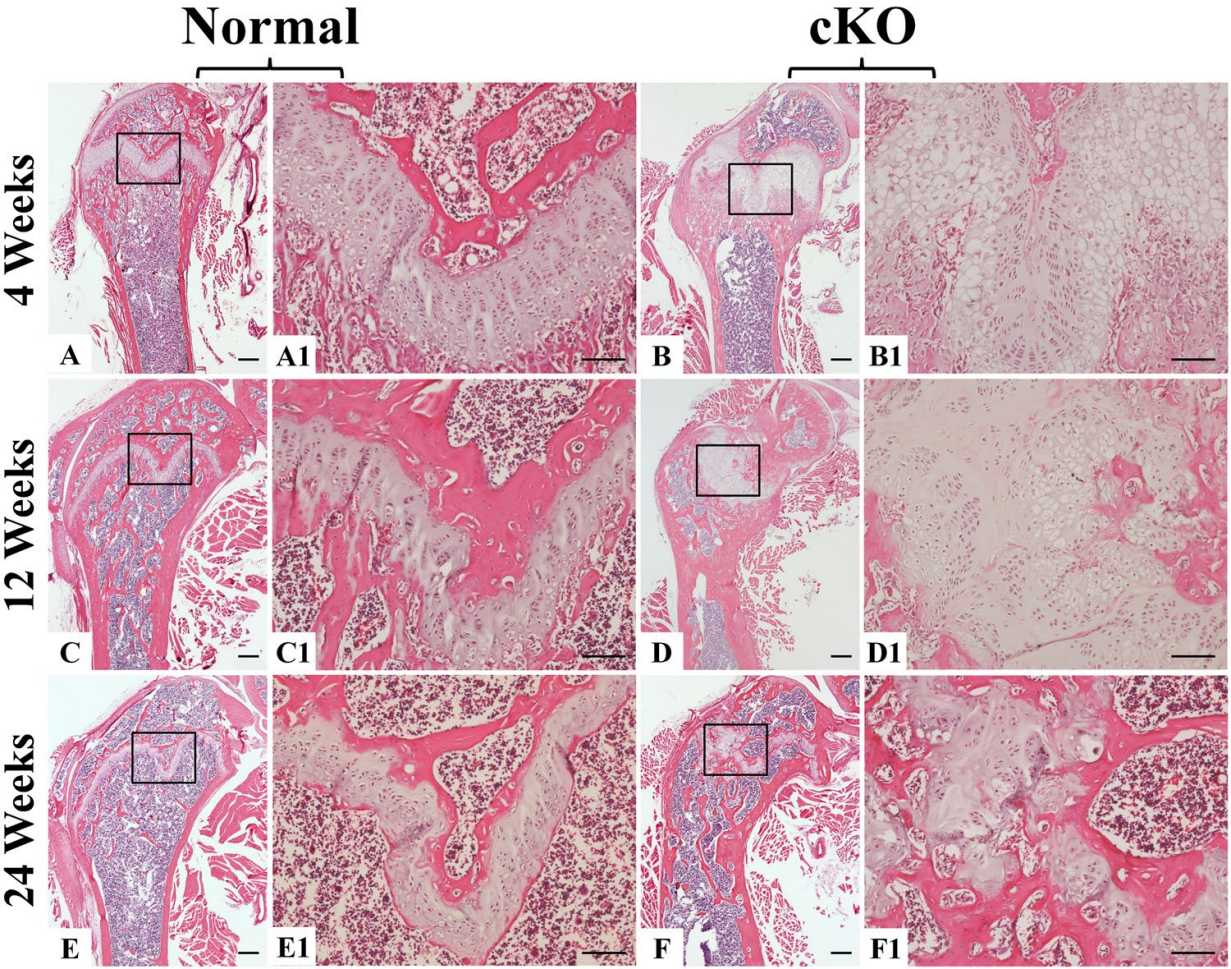
H&E staining of femurs from 4-, 12- and 24-week-old mice. At postnatal 4 and 12 weeks, the growth plates in the cKO mice were much wider than in the normal mice; different segments of the growth plates in the cKO mice showed great variations in thickness. (**A**,**A1**,**B**,**B1**,**C**,**C1**,**D**,**D1**). In particular, the hypertrophic zone of growth plate in the 4-week-old cKO mice appeared dramatically enlarged (**B**,**B1**). At postnatal 24 weeks, the growth plate in the cKO mice was disorganized compared to the normal mice (**E**,**E1**,**F**,**F1**). (**A1**,**B1**,**C1**,**D1**,**E1** and **F1**) were the higher magnification views of the box areas in (**A**,**B**,**C**,**D**,**E** and **F**) respectively. Bars in (**A**,**B**,**C**,**D**,**E** and **F**) 500 µm; bars in (**A1**,**B1**,**C1**,**D1**,**E1** and **F1**) 100 µm.

X-ray radiography revealed that the zones of growth plates in the cKO mice (arrows in Fig. 2B,D) were much wider than in the normal mice. Histological examination showed that at postnatal 4 weeks, the growth plate cartilage in the normal mice had a rather uniform thickness across the plate (Fig. 3A,A1) whereas different segments of the growth plate in the cKO mice displayed great variations in thickness (Fig. 3B,B1). In particular, the hypertrophic zone of growth plate in the cKO mice appeared dramatically enlarged (Fig. 3B,B1). The growth plates in 12- and 24-week-old normal mice were narrower than in the 4-week-old normal mice (Fig. 3C,C1,E,E1). The growth plates of the 12- and 24-week-old cKO mice were disorganized and showed a general increase in thickness (Fig. 3D,D1,F,F1) compared to the normal mice.

### The cortical bone of cKO mice had more osteoid, abnormal collagen structure, and altered mineral deposition

**Figure 4:**
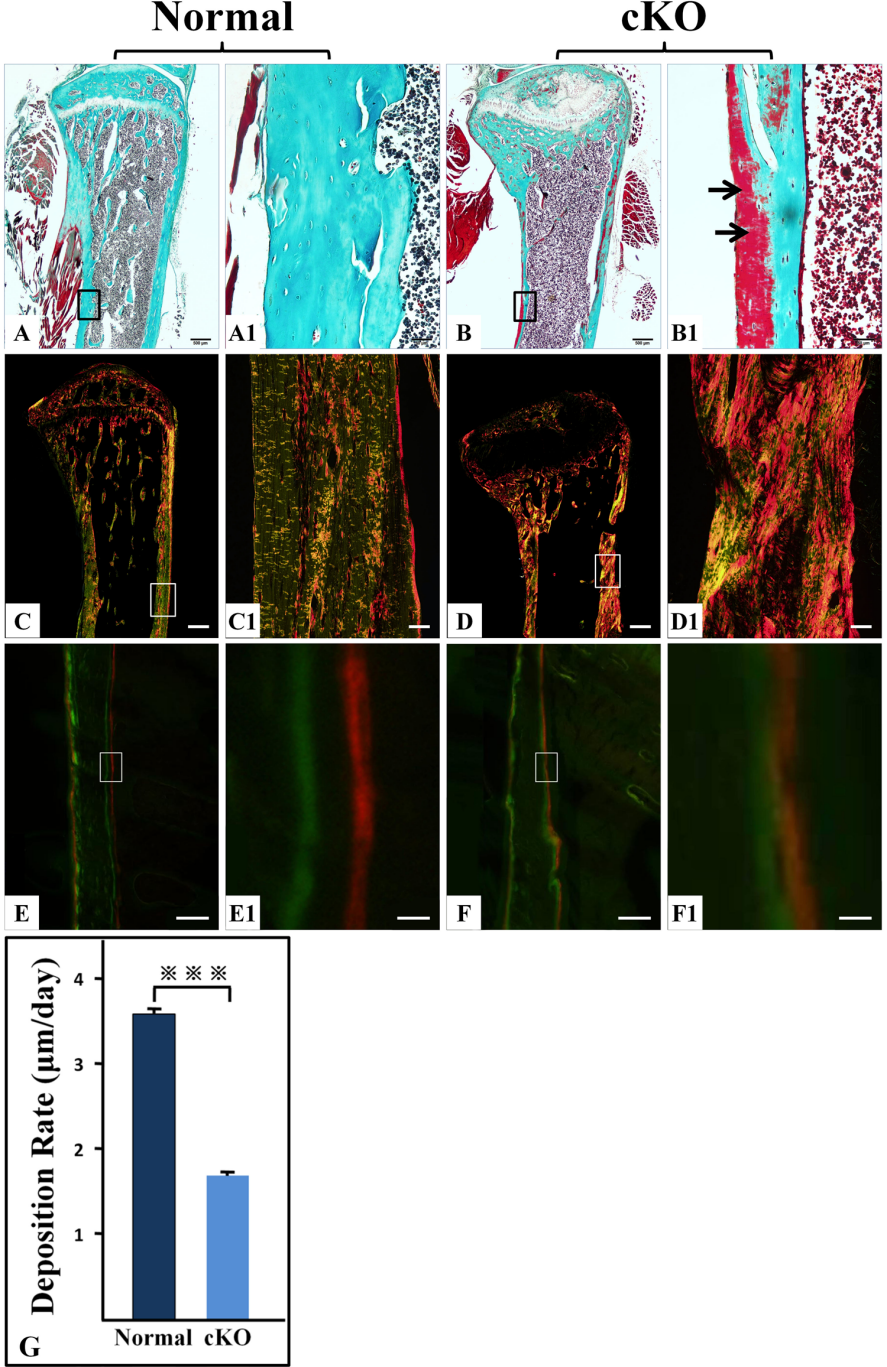
Goldner’s Masson trichrome staining, picro-sirius red staining, and double fluorochrome labeling. (**A**–**B1**) Goldner’s Masson trichrome staining of tibias from 12-week-old mice. (**C**–**D1**) picro-sirius red staining of tibias from 12-week-old mice. (**E**–**F1**) double fluorochrome labeling of femurs from 4-week-old mice. (**G**) quantitative analyses of data acquired from the double fluorochrome labeling specimens from five mice (n  = 5); ***P < 0.001. (**A1**,**B1**,**C1**,**D1**,**E1** and **F1**) were the higher magnification views of the box areas in (**A**,**B**,**C**,**D**,**E** and **F**), respectively. Bars in (**A**,**B**,**C** and **D**) 500 µm; bars in (**A1**,**B1**,**C1** and **D1**) 50 µm; bars in (**E** and **F**) 100 µm; bars in (**E1** and **F1**) 10 µm. Goldner’s Masson trichrome staining of tibias in 12-week-old normal (**A**,**A1**) and cKO mice (**B**,**B**1) showed that the cKO mouse bone had large areas stained red, indicating an accumulation of osteoid in the *Fam20C*-deficient bone (arrows in **B1**). Compared to the 12-week-old normal mice (**C**,**C1**), the collagen fibers in the femoral cortical bone of the cKO mice (**D**,**D1**) appeared disorganized and thicker. The changes in the double fluorochrome labeling appeared to be mainly in the endosteal region of femurs in the 4-week-old cKO mice (**F**,**F1**).

In Goldner’s Masson trichrome staining, the well-mineralized tissues are stained green while the hypomineralized or non-mineralized tissues such as osteoid are stained red. The cortical bone in the tibias of 12-week-old normal mice was stained uniformly green (Fig. 4A,A1), while that of the cKO mice (Fig. 4B,B1) had large areas stained red indicating an excessive accumulation of osteoid in the *Fam20C*-deficient bone (arrows in Fig. 4B1).

To assess the collagen structure of the cortical bone, we performed picro-sirius red staining to visualize the collagen fibers in the cortical bone of tibias from 12-week-old mice. When examined under polarized light, the larger collagen fibers in picro-sirius red staining are bright yellow or orange, and the thinner ones, including reticular fibers, are green. Compared to the normal mice (Fig. 4C,C1), the collagen fibers in the femoral cortical bone of the cKO mice (Fig. 4D,D1) appeared disorganized and thicker.

Double fluorochrome labeling analyses revealed that the cortical bone of femurs from 4-week-old normal mice showed two distinct layers of labeling that were separated by a significant distance (Fig. 4E,E1), while in the cKO mice the green and red zones were quite close with incomplete separation in certain regions suggesting a reduced and disturbed deposition of calcium in the *Fam20C*-deficeint bone (Fig. 4F,F1). Quantitative analyses of the double labeling specimens from 5 mice in each group (n = 5) showed that the calcium deposition rate calculated from the average distance between the green and red zones was 3.5 µm/day in the normal mice and 1.7 µm/day in the cKO mice (Fig. 4G). The difference in the calcium deposition rate between the cKO and normal mouse bone was statistically significant (P < 0.001). The reduction in mineral deposition of cKO mouse bone was consistent with the observations from the Goldner’s Masson trichrome staining and X-ray analyses that the cKO mice had increased amounts of osteoid, less volume of mineralized bone and lower level of mineralization in the long bone.

### Altered levels of extracellular matrix proteins in the long bone of cKO mice

**Figure 5:**
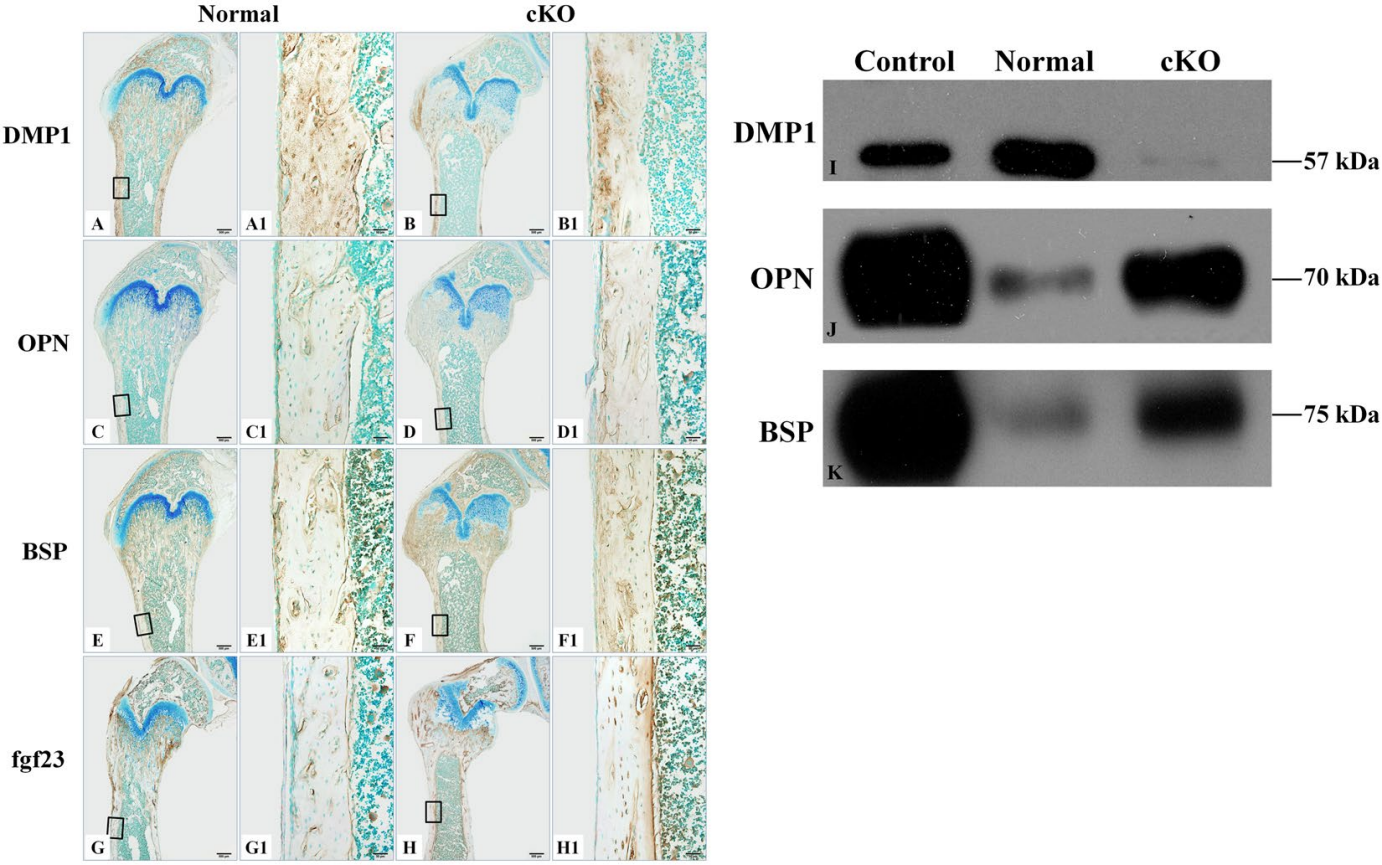
Assessment of ECM protein levels in the cKO mice versus normal mice. (**A**–**H1**) immunohistochemistry for DMP1 (**A–B1**), OPN (**C–D1**), BSP (**E–F1**) and FGF23 (**G–H1**) in the femurs of 4-week-old mice; (**A1**,**B1**,**C1**,**D1**,**E1**,**F1**,**G1** and **H1**) were the higher magnification views of the box areas in **A,B,C,D,E,F,G** and **H**, respectively. (**A**,**B**,**C**,**D**,**E**,**F**,**G** and **H**) were from consecutive sections. Bars in (**A**,**B**,**C**,**D**,**E**,**F**,**G** and **H**): 500 µm; bars in (**A1**,**B1**,**C1**,**D1**,**E1**,**F1**,**G1** and **H1**) 50 µm. (**I–K**) Western immunoblotting analyses for DMP1, OPN and BSP in the protein extracts from the long bone of 12-week-old mice; equal amounts of total proteins from the normal and cKO groups were loaded for detecting each of the proteins. The cortical bone of the cKO mice (**B**,**B1**) contained many areas that lacked anti-DMP1 signals. Immunohistochemistry analyses showed that the levels of OPN, BSP and FGF23 were higher in the cortical bone of the cKO mice than in the normal mice (**C**,**C1**,**D**,**D1**,**E**,**E1**,**F**,**F1**,**G**,**G1**,**H**,**H1**). In the Western immunoblotting analyses, DMP1 band in the cKO group was weaker while that of OPN or BSP was stronger than in the normal mice (**I**,**J**,**K**). The samples in the control lanes were DMP1, OPN and BSP, respectively; these proteins were extracted and purified from the rat long bone^35^.

Immunohistochemistry staining performed on the femurs of 4-week-old mice revealed that compared to the normal mice (Fig. 5A,A1), DMP1 was reduced in the cortical bone of the cKO mice (Fig. 5B,B1); many areas of the cortical bone in the cKO mice showed very faint or no anti-DMP1 signals. On the contrary, the levels of OPN and BSP were higher in the cortical bone of the cKO mice than in the normal mice (Fig. 5C,C1,D,D1,E,E1,F,F1). The cortical bone of normal mice showed weak to moderate anti-FGF23 immunoreactivity, primarily in and around the osteocytes (Fig. 5G,G1). The cortical bone of the cKO mice (Fig. 5H,H1) demonstrated much stronger signals for FGF23 than the normal mice. Western immunoblotting analyses further confirmed that the long bone matrix of cKO mice hag much less DMP1 and more OPN or BSP than that of the normal mice (Fig. 5I,J,K).

### Loss of FAM20C led to elevation of serum FGF23 and reduction of serum phosphorus

**Figure 6:**
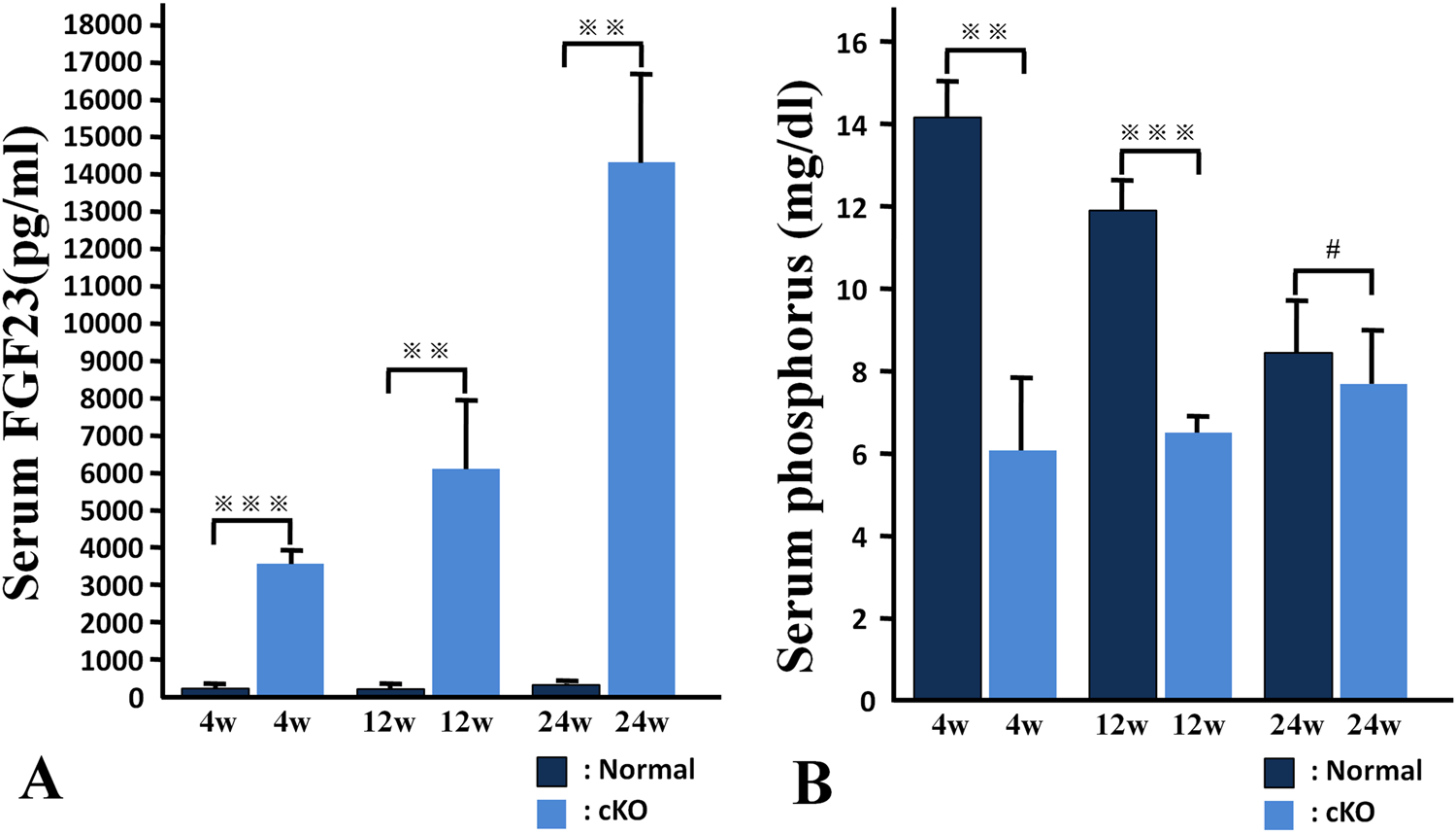
Serum FGF23 and phosphorus (Pi) levels. **P < 0.05; ***P < 0.01; ^#^P > 0.05. The serum FGF23 level was remarkably higher in the cKO mice than the normal mice at each of the time points (**A**). The serum phosphorus levels in the 4-week-old and 12-week-old cKO mice were significantly lower than in the age-matched normal mice. The serum phosphorus level in 24-week-old cKO mice was not statistically different from the normal mice (**B**).

We measured the FGF23 and phosphorus levels in the sera of 4-, 12- and 24-week-old mice. The circulating FGF23 level was remarkably elevated in the cKO mice at all of the three observation points (Fig. 6A). The increase in serum FGF23 levels in the cKO mice became more remarkable as the animal aged. The serum FGF23 level in 24-week-old cKO mice was approximately 3.9 times of that in 4-week-old cKO mice, and 2.3 times of that in 12-week-old cKO mice. The serum phosphorus levels in the 4-week-old and 12-week-old cKO mice were significantly lower than those of the normal mice at the same ages (Fig. 6B). Interestingly, the serum phosphorus level of 24-week-old cKO mice was only slightly lower than in the age-matched normal mice, and the phosphorus reduction in the 24-week-old cKO mice was not statistically significant compared to the normal mice of the same age.

## Discussion

FAM20C is expressed by a variety of cells including the cells forming the mineralized tissues and those present in non-mineralized tissues and body fluids^1, 16, 17^. *In vitro* studies revealed that as a protein kinase, FAM20C is able to catalyze the attachment of phosphates to more than a hundred types of proteins including those present in the milk, blood and other body fluids, as well as the proteins secreted into the extracellular matrices^2–6^. The ubiquitous distribution and broad substrate spectrum of FAM20C suggest that in addition to its role in biomineralization, this kinase may participate in lipid homeostasis, wound healing, cell differentiation, cell migration and cell adhesion^3^. The broad spectrum of FAM20C functions speculated for different tissues and cells may account for the heterogeneous manifestations of human Raine syndrome caused by *FAM20C* mutations^7–12^. Therefore, there is a necessity to nullify FAM20C in a specific type of cells in order to analyze the biological role of this enzyme in individual tissues or organs. This investigation showed that nullifying *Fam20C* in the cells expressing type I collagen led to skeletal defects and a reduction of serum phosphorus. The phenotypic changes in the skeletons and serum of the *2.3* *kb Col 1a1-Cre;Fam20C* ^*flox/flox*^ mice were consistent with the diagnosis of hypophosphatemic rickets.

While the hypophosphatemic rickets phenotypes of *2.3* *kb Col 1a1-Cre;Fam20C* ^*flox/flox*^ mice, to a certain degree, resembled those of the *Sox-Cre;Fam20C* ^*flox/flox*^ mice in which *Fam20C* was inactivated in nearly all the tissues, the general health of the former was better than the later. The body weight of *2.3* *kb Col 1a1-Cre;Fam20C* ^*flox/flox*^ mice at postnatal 6 weeks was approximately 180% of the *Sox-Cre;Fam20C* ^*flox/flox*^ mice at the same age. Such observations support the speculations drawn from *in vitro* studies that in addition to its participation in biomineralization, FAM20C play very broad roles in a variety of tissues and cells^3^. Nevertheless, the fact the major abnormalities in the *Sox-Cre;Fam20C* ^*flox/flox*^ mice occur in the mineralized tissues^13, 14^ highly suggests that the most important functions of FAM20C may lie in the formation of skeletal and dental tissues. In other words, FAM20C’s roles in phosphorylating proteins secreted into the matrices of bone, dentin, enamel and cementum are likely to be essential, while its kinase functions in certain non-mineralizing tissues perhaps can be compensated to a certain extent by other molecules. Clearly, there is a need to specifically inactivate FAM20C in other types of cells such as the brain cells, blood cells or muscle cells.

Chondrocytes in the growth plate express a significant level of FAM20C^16^ and the *Sox-Cre;Fam20C* ^*flox/flox*^ mice had defective growth plate suggesting that FAM20C may play certain roles in chondrogenesis^13^. Chondrocytes in the growth plate do not express type I collagen and *Fam20C* was still active in the chondrocytes of the growth plate in *2.3* *kb Col 1a1-Cre;Fam20C* ^*flox/flox*^ mice. Nevertheless, the *2.3* *kb Col 1a1-Cre;Fam20C* ^*flox/flox*^ mice analyzed in the present study had disorganized and widened growth plate. We postulate that the growth plate defects in the *2.3* *kb Col 1a1-Cre;Fam20C* ^*flox/flox*^ mice or perhaps also the abnormal growth plates observed in the *Sox-Cre;Fam20C* ^*flox/flox*^ mice may be due to systemic factors such as high level of serum FGF23^18, 19^ and lower level of serum phosphorus^20, 21^. Another speculation regarding the changes in the growth plate of the *2.3* *kb Col 1a1-Cre;Fam20C* ^*flox/flox*^ mice in which *Fam20C* was not ablated in chondrocytes may be related to potential interacting relationships between the chondrocytes and endochondral bone formation. Several recent studies have indicated that in the process of endochondral bone formation of the long bones, chondrocytes may directly differentiate into osteogenic cells^22–25^. If a certain population of chondrocytes in the growth plate are transformed into osteoblasts that are responsible for new bone formation, then it is tempting to speculate that when the endochondral bone formation is defective, the osteogenic cells may send feedback signals via an unknown signaling pathway to the chondrocytes in the growth plate and may affect the normal development of the cartilaginous tissues, leading to the growth plate defects in the long bones of the *2.3* *kb Col 1a1-Cre;Fam20C* ^*flox/flox*^ mice.

Although other types of cells secrete FGF23, osteoblasts and osteocytes are the major sources of FGF23 production^26–28^. Elevation of serum FGF23 is known to increase phosphate wasting in the kidney, which may lead to hypophosphatemia^26, 29–31^. In a previous study on the *Sox-Cre;Fam20C* ^*flox/flox*^ mice, we showed elevation of serum FGF23 and reduction of serum phosphorus in the *Fam20C*-deficient mice; in that previous investigation we only examined the serum FGF23 and phosphorus levels in 6-week-old mice and did not analyze their progressive changes in relevance to the increase of age^13^. In the current study, we measured the serum FGF23 and phosphorus levels in the mice at the ages of postnatal 4, 12 and 24 weeks. Our data showed that the elevation of serum FGF23 level became more remarkable as the cKO mice aged; the serum FGF23 level in 24-week-old cKO mice was approximately 3.9 times of that in 4-week-old cKO mice and 2.3 times of that in 12-week-old cKO mice. The increase of serum FGF23 with age in the cKO mice may be due to the continuous accumulation of the full-length FGF23 with GalNAc-T3 O-glycosylation of Thr^178^, which is believed to prevent the proteolytic cleavage and degradation of this protein^32^. Thus, as the cKO animals age, more and more full-length FGF23, which was not cleavable due to O-glycosylation of Thr^178^, accumulates in the circulation. Interestingly, the reduction of serum phosphorus level did not follow the elevation pattern of serum FGF23 in a proportional manner. The fact the serum phosphorus reduction in the cKO mice did not correlate proportionally to the elevation of serum FGF23 suggests that the hypophosphatemia in the *Fam20C*-deficeint subjects may not be fully dependent on the elevation of circulating FGF23. In addition to serum FGF23 elevation, other factors may also contribute to the hypophosphatemic phenotype in the *Fam20C*-deficeint subjects. The serum phosphorus level in 24-week-old cKO mice was slightly lower than the normal mice of the same age, and the change was not statistically significant, indicating that as the animals aged, hypophosphatemia was compensated; at this point, we do not have a clear explanation regarding the compensation changes of serum phosphorus level in the aged animals.

In this study, we observed a reduction of DMP1 in the extract of long bone from the cKO mice, which may be associated with the pathogenesis of skeletal defects caused by the loss-of-function of *Fam20C*. DMP1 is a substrate of FAM20C^2, 6^. Without FAM20C, DMP1 cannot be properly phosphorylated, which may lead to the loss of its functions. When DMP1 cannot be phosphorylated, osteoblasts and osteocytes may down-regulate this protein in order to avoid wasting products, or perhaps when this protein cannot be phosphorylated, its degradation is accelerated, leading to a reduction in its protein level. The skeletal phenotypes in *Fam20C*-cKO mice also showed certain similarities to the *Dmp1*-deficient animals^33, 34^. Taken together, we postulate that the lack of proper phosphorylation in and/or the reduction of DMP1 may be one of the contributing factors in causing the rachitic phenotypes in the *Fam20C*-cKO mice and in some human patients with Raine syndrome. In contrast, OPN level increased in the bone of *Fam20C*-cKO mice. OPN is a potent inhibitor of mineralization^35, 36^. Elevation of OPN in the bone ECM may accelerate the rachitic defects of the cKO mice as in the case of FGF23-deficient mice^37^. Taken together, the reduction of DMP1 and elevation of OPN in the matrix of bone may be aggravating factors in the development of rickets in FAM20C-defcient subjects.

## Methods

### Generation of 2.3 kb Col 1a1-Cre;Fam20C^*flox*/*flox*^(cKO) mice

The *2.3* *kb Col 1a1-Cre;Fam20C* ^*flox/flox*^ (cKO) mice were generated by breeding the *2.3* *kb Col 1a1-Cre* mice^38^ with the *Fam20C* ^*flox/flox*^ mice^13^. The genotyping approaches and validation of FAM20C inactivation in type I collagen-expressing cells of cKO mice have been described in our previous report^15^. The *Fam20C* ^*flox/flox*^ mice from the same litters as the cKO mice created during the crossbreeding regime were used as normal controls; utilizing the *Fam20C* ^*flox/flox*^ littermates of cKO mice as normal controls not only reduced the number of mice needed but also prevented potential variances that might result from comparing mice out of different litters. Both male and female mice were used in the study, and we did not observe significant phenotypic differences between the male and female cKO mice.

All animal procedures were approved by the Institutional Animal Care and Use Committee (IACUC) of Texas A&M University Baylor College of Dentistry. All experiments were performed in accordance with the guidelines and regulations issued by the USA National Institutes of Health regarding the use of animals and biological reagents.

### Gross observation and Alizarin red/Alcian blue staining

The mice were weighed every week from postnatal 3 weeks until postnatal 12 weeks to monitor the animal growth; the same animals (not sacrificed before postnatal 12 weeks) were weighed at different time points. The average values of the body weight calculated from 10 mice at each age point were used to make growth curves. Alizarin red/Alcian blue staining of the skeletons was performed on 1-week-old normal and cKO mice to visualize the skeleton and the overall mineralization levels, as we previously described^13^.

### X-ray radiography

The tibias dissected from the normal and cKO mice at postnatal 4, 12 and 24 weeks were analyzed using plain X-ray radiography (Faxitron MX-20DC12 system; Faxitron Bioptics, Tucson, Arizona, USA). The femurs dissected from these mice were examined by μCT radiography (Scanco μCT35 imaging system; Scanco Medical, Brüttisellen, Switzerland) using a low-resolution scan (12-µm slice increment) for morphological observations, as we previously reported^15^. The data acquired from the high-resolution scans (3.5-µm slice increment) of the midshaft regions from the femurs of five 12-week-old mice (n = 5) were used for quantitative analyses to reveal the ratio of Bone Volume to Total Volume (BV/TV), Apparent Density and Material Density. The quantitative data were reported as mean ± SD and analyzed by Student’s t test. Changes with P < 0.05 were considered statistically significant in the quantitative analyses.

### Preparation of decalcified sections, and hematoxylin and eosin (H&E) staining

The femurs and tibias from 4-, 12- and 24-week-old mice were fixed overnight at 4 °C with 4% paraformaldehyde in phosphate buffered saline (PBS) solution and then decalcified in 15% ethylenediaminetetraacetate (EDTA) solution (pH 7.4) at 4 °C for 5~14 days, depending on the ages of the animals. The samples were processed for paraffin embedding, and 5-µm serial sections were prepared for H&E staining, picro-sirius red staining, and immunohistochemistry analysis.

### Goldner’s Masson trichrome staining, picro-sirius red staining, and double fluorochrome labeling

The undecalcified sagittal sections of 10 µm thickness from the tibias of 12-week-old mice were stained using Goldner-Masson trichrome assay, as we previously described^13^. The cortical bone areas of the diaphysis were photographed. In the Goldner’s Masson trichrome staining the unmineralized osteoid is stained red/orange, and mineralized bone is stained green/blue.

For picro-sirius red staining, the decalcified sections from tibias of 12-week-old mice were immersed in hematoxylin solution for 8 minutes to stain the nuclei and washed for 10 minutes in water. The sections were then stained in picro-sirius red for one hour, washed in two changes of acidified water, dehydrated in three changes of 100% ethanol, cleared in xylene and mounted. We analyzed the structure and organization of collagen fibers in the tibias under polarized light microscopy.

Double fluorochrome labeling was performed as we previously described^13^. Briefly, calcein green (10 mg/kg Sigma-Aldrich) was administered to the 3-week-old mice through intraperitoneal injection. Five days later, Alizarin red (30 mg/kg, Sigma-Aldrich) was injected. The mice were sacrificed 48 hours after the intraperitoneal injection of Alizarin red and the femurs were embedded in methylmethacrylate (MMA) and then, 10 µm of undecalcified sections were prepared. The unstained section was observed under fluorescent microscope. Five mice from each group (n = 5) were used for the double fluorescence labeling analyses. The distances between the green and red zones were measured and calculated to estimate the calcium deposition rate. The data from the quantitative analyses were reported as mean ± SD and analyzed by Student’s t test. The changes with P < 0.05 were considered statistically significant.

### Immunohistochemistry (IHC) staining

The IHC experiments on paraffin-embedded sections were carried out using ABC kit and DAB kit (Vector Laboratories, Burlingame, California, USA) according to the manufacturer’s instructions. We employed polyclonal antibodies against DMP1^39^, OPN^40^ and BSP (LF87, a gift from Dr. Larry Fisher of National Institute of Dental and Craniofacial Research), to detect these SIBLING family members as we previously reported^15^. A monoclonal antibody against FGF23 (anti-FGF23-79)^41^ was used to detect this protein in the mouse long bone. In the IHC analyses, for each type of antibodies, the specimens from the normal and cKO mice from the same litters were stained in the same batch of experiments to ensure that exactly same conditions were applied to the different groups. The same concentrations of normal rabbit serum or rabbit IgG were used to replace the primary antibodies serving as negative controls for the IHC experiments detecting DMP1, OPN and BSP. The same concentration of mouse IgG was used to replace the anti-FGF 23 antibody, acting as a negative control for this monoclonal antibody. The IHC sections were counterstained with methyl green.

### Western immunoblotting

Western immunoblotting was performed to evaluate levels of DMP1, OPN and BSP in the total extracts of non-collagenous proteins from the long bones of 12-week-old mice. The non-collagenous proteins were extracted from the mouse leg bones as we previously reported^39^. The protein concentrations of the samples were measured using the BicinchoninicAcid (BCA) assay (Pierce Biotechnology). For anti-DMP1 immunoblotting, 5 µg of the protein extracts from either the normal control or the cKO group was loaded onto 5–15% sodium dodecyl sulfatepolyacrylamide gel electrophoresis (SDS-PAGE) gels. For anti-OPN immunoblotting, 0.05 µg of the sample was used. For anti-BSP Western immunoblotting, 0.5 µg of the protein extracts was loaded. The protein bands in the SDS-PAGE gels were transferred onto polyvinylidene difluoride (PVDF) membrane (BIO-RAD). The protein-containing membrane was incubated with the above-described anti-DMP1, anti-OPN and anti-BSP antibodies at 4 °C for overnight. The goat-derived anti-rabbit IgG antibodies conjugated with horseradish peroxidase (Santa Cruz Biotechnology, Dallas, TX; 1: 4000) were used as the secondary antibodies. The immunoreactive bands were detected with an Enhanced Chemiluminescence (ECL) detection system (Pierce Biotechnology), according to the manufacturer’s instructions. Chemiluminescent bands were imaged using CL-XPosure film (Pierce Biotechnology Inc., Rockford, Ill., USA).

### Measurements of FGF23 and phosphorus in the serum

The serum FGF23 levels were measured using a full-length FGF23 ELISA kit (Kainos Laboratories, Japan). Serum phosphorus was measured using the phosphomolybdate-ascorbic acid method, as we previously described^13^. The data acquired from the sera of 5 mice (n  = 5) for each age group were used for comparative and statistical analyses. The data were reported as mean ± SD and analyzed by Student’s t test. The changes with P < 0.05 were considered statistically significant.
